# Mononuclear cells and vascular repair in HHT

**DOI:** 10.3389/fgene.2015.00114

**Published:** 2015-03-23

**Authors:** Calinda K. E. Dingenouts, Marie-José Goumans, Wineke Bakker

**Affiliations:** Department of Molecular Cell Biology, Leiden University Medical CenterLeiden, Netherlands

**Keywords:** homing, myocardial ischemia and infarction, TGF-beta, dipeptidyl peptidase 4, mononuclear cells, regenerative medicine, tissue therapy, cardiovascular disease

## Abstract

Hereditary hemorrhagic telangiectasia (HHT) or Rendu–Osler–Weber disease is a rare genetic vascular disorder known for its endothelial dysplasia causing arteriovenous malformations and severe bleedings. HHT-1 and HHT-2 are the most prevalent variants and are caused by heterozygous mutations in endoglin and activin receptor-like kinase 1, respectively. An undervalued aspect of the disease is that HHT patients experience persistent inflammation. Although endothelial and mural cells have been the main research focus trying to unravel the mechanism behind the disease, wound healing is a process with a delicate balance between inflammatory and vascular cells. Inflammatory cells are part of the mononuclear cells (MNCs) fraction, and can, next to eliciting an immune response, also have angiogenic potential. This biphasic effect of MNC can hold a promising mechanism to further elucidate treatment strategies for HHT patients. Before MNC are able to contribute to repair, they need to home to and retain in ischemic and damaged tissue. Directed migration (homing) of MNCs following tissue damage is regulated by the stromal cell derived factor 1 (SDF1). MNCs that express the C-X-C chemokine receptor 4 (CXCR4) migrate toward the tightly regulated gradient of SDF1. This directed migration of monocytes and lymphocytes can be inhibited by dipeptidyl peptidase 4 (DPP4). Interestingly, MNC of HHT patients express elevated levels of DPP4 and show impaired homing toward damaged tissue. Impaired homing capacity of the MNCs might therefore contribute to the impaired angiogenesis and tissue repair observed in HHT patients. This review summarizes recent studies regarding the role of MNCs in the etiology of HHT and vascular repair, and evaluates the efficacy of DPP4 inhibition in tissue integrity and repair.

## HHT and the Underlying Genetic Causes

Hereditary hemorrhagic telangiectasia (HHT) or Rendu–Osler–Weber disease is a genetic vascular disorder. The onset of the disease and severity is variable for each individual patient and will intensify as the disease progresses with age (Plauchu et al., 1989). To be diagnosed with HHT, a patient has to have 3 of the 4 Curaçao criteria, namely epistaxis, telangiectasias, arteriovenous malformations (AVMs), or a first degree relative with HHT. After diagnosis, patients are genetic screened to identify the underlying mutation (Shovlin et al., 2000). Since the underlying mutation cannot always be identified in every patient, the clinical symptoms are still important for the diagnosis of HHT. To date, four genes were found to be mutated, resulting in four different HHT subtypes. All genes identified are components of the transforming growth factor beta (TGFβ) signaling pathway. The identified mutations will not generate aberrant proteins, but will rather result in haploinsufficiency, a reduction of the functional protein levels by 50%, causing a disbalance in the TGFβ signaling pathway (Bourdeau et al., 2000; Abdalla and Letarte, 2006). HHT-1 is the most prevalent HHT subtype, comprising 53% of Dutch HHT patients (Letteboer et al., 2008). The HHT-1 mutation lies in the endoglin gene (McAllister et al., 1994), a TGFβ co-receptor modulating TGFβ and BMP signaling and crucial for angiogenesis and vascular repair (Pardali et al., 2010). The second most prevalent gene found to be mutated is the activin receptor-like kinase 1 (ALK1) and causes HHT-2 (Johnson et al., 1996). Approximately 40% of the Dutch HHT patients have this HHT-2 variant (Letteboer et al., 2008). ALK1 is a type I receptor able to signal downstream of either BMP or TGFβ, depending on the ligand availability and receptor context (Goumans et al., 2009). Hundreds of variants have been described for both HHT-1 and HHT-2, amounting to ∼87% of HHT cases globally, leaving about 15–20% of HHT families without a characterized mutation (Garg et al., 2014). HHT-3 and HHT-4 are linked to loci on chromosomes 5 and 7 respectively, but the exact genes affected are not yet identified (Cole et al., 2005; Bayrak-toydemir et al., 2006). The third and fourth gene in which mutations are found causing HHT are BMP9 and SMAD4. Interestingly, BMP9 is a ligand for ALK1 (Wooderchak-Donahue et al., 2013) and SMAD4 is a transcription factor involved in transducing BMP and TGFβ signals from the cell membrane into the nucleus. Mutations in SMAD4 cause a combined syndrome of HHT and juvenile polyposis (Gallione et al., 2006).

In this review the consequences of a disturbed TGFβ signaling cascade caused by the different mutations found in HHT will be described, especially how this affects mononuclear cell (MNC) functioning and their capacity to repair.

## Impaired Angiogenesis in HHT is Caused by Disrupted TGFβ Signaling

As mentioned above, all genes that have been found mutated in HHT are linked to TGFβ signaling. Upon tissue damage, TGFβ is released by the extracellular matrix, apoptotic cells or secreted by platelets, macrophages and T lymphocytes (Grainger et al., 2000; Wan et al., 2012). TGFβ is the prototypic member of a large superfamily to which also activin and BMPs belong. To be able to signal, TGFβ ligands bind to the TGFβ receptor type II, and BMP ligands can to bind to both the BMP receptor types I and II (Goumans et al., 2009). Upon binding of the ligand, a TGFβ type I receptor is recruited and a heterotetrameric complex is formed, which in turn phosphorylates intracellular receptor regulated SMAD proteins. In endothelial cells, TGFβ can signal using two type I receptors, namely via ALK5 resulting in the phosphorylation of SMAD 2 and 3, or by the BMP type I receptor, ALK1 followed by activation of SMAD 1 and 5. ALK1 can only form a complex with the TGFβ receptor type II in the presence of ALK5 in the tetrameric complex and the presence of endoglin as co-receptor (Lebrin et al., 2004; Goumans et al., 2009).

The presence of these two pathways might explain the biphasic effect TGFβ has on angiogenesis, since TGFβ-ALK1 signaling induces endothelial cell proliferation and migration, whereas TGFβ-ALK5 signaling leads to a quiescent endothelium. Endoglin mainly stimulates the TGFβ-ALK1 pathway, and is thought to suppress TGFβ-ALK5 signaling (Goumans et al., 2008). After phosphorylation, the receptor regulated SMADs form a complex with SMAD4, and translocate into the nucleus where they act as a transcription factor to ensure target gene expression.

Since endoglin is involved in endothelial cell proliferation, migration, and remodeling of the extracellular matrix (Lebrin et al., 2004; Abdalla and Letarte, 2006), the vascular defects and impaired angiogenesis observed in HHT-1 are largely explained by malfunctioning of the endothelial cells (Jerkic et al., 2006; Düwel et al., 2007; Liu et al., 2014). However, although highly expressed on activated endothelial cells, endoglin is also present on stromal cells, smooth muscle cells, mesenchymal and hematopoietic stem cells, and MNC (Kapur et al., 2013). The importance of endoglin for endothelial cell homeostasis became evident when endothelial cells that lack one allele of endoglin were studied. Endoglin heterozygous endothelial cells exhibit reduced ALK1-Smad1/5 signaling. Unexpectedly, these cells adapted their ALK5 expression with a decrease of 80% and therefore also have reduced ALK5-Smad2/3 signaling (Lebrin et al., 2004; Lebrin and Mummery, 2008). In contrast, endoglin deficient endothelial cells show an increased ALK1 and ALK5 signaling (Pece-Barbara et al., 2005). This demonstrates that endoglin haploinsufficiency affects downstream TGFβ signaling and gene adaptation. When comparing different studies, we could conclude that the mutations underlying the various HHT subtypes converge in the ALK1 arm of the TGFβ pathway; affecting endoglin, ALK1, BMP9, and SMAD4 proteins. The imbalance caused by the haploinsufficiency of these proteins skews TGFβ signaling toward endothelial cell quiescent state, leading to impaired angiogenesis after tissue injury.

Another manifestation in HHT is the formation of weak blood vessels, as a result of impaired maturation. Lebrin et al. (2010) found that the anti-angiogenic drug thalidomide induces the recruitment of mural cells such as pericytes and vascular smooth muscle cells. The recruitment of these cells to vessel branching points enhanced the maturation of HHT vessels and reduced the occurrence of epistaxis (Lebrin et al., 2010). Unfortunately thalidomide treatment is prone to side effects such as peripheral neuropathy and fatigue (Ghobrial and Rajkumar, 2003; Morawska and Grzasko, 2014). Current research is focused on finding a compound with similar mode of action, restoring the maturation of the diseased blood vessels.

## Unraveling the Etiology and Mechanism Behind HHT: Lessons from Mouse Models

Murine models have given valuable insights into the mechanism behind the mutations found in HHT patients. The different heterozygous mouse models confirmed that the defect in TGFβ signaling due to the haploinsufficiency resembled HHT, as they developed similar vascular abnormalities like telangiectasias, AVMs and endothelial dysplasia (Lowery and de Caestecker, 2010). While endoglin deficient mice are embryonically lethal around embryonic day (E)10.5 and show defects in cardiac development and impaired maturation of blood vessels in the yolk sac (Bourdeau et al., 1999; Arthur et al., 2000), endoglin heterozygous mice are vital. However, adult endoglin heterozygous mice show impaired angiogenesis, AVMs and display cerebral vascular abnormalities (Satomi et al., 2003; van Laake et al., 2006). Choi et al. (2014) specifically deleted endoglin in endothelial and smooth muscle cells using the SM22α-Cre mouse model. Combined with local VEGF stimulation in the brain, this endoglin deletion causes cerebral AVMs (Choi et al., 2014). However, VEGF stimulation together with deletion of endoglin in the endothelium alone is already enough to cause vascular dysplasia (Choi et al., 2012).

Activin receptor-like kinase 1 deficient mice are also embryonically lethal on E10.5 due to severe hematopoietic defects, AVMs and impaired angiogenesis in the embryo as well as in the yolk sac (Oh et al., 2000; Urness et al., 2000; Sorensen et al., 2003). ALK1 heterozygous mice are viable and display HHT-2 like symptoms, such as vascular malformations, lesions, and hemorrhages (Srinivasan et al., 2003). Interestingly, endothelial cell specific ALK1 deletion leads to the formation of retinal AVMs and pulmonary hemorrhages, but also causes a reduced expression of endoglin (Tual-Chalot et al., 2014).

Since SMAD4 null mice are embryonically lethal at day 7, and SMAD4 heterozygous mice show no abnormalities, developing a mouse model for this subtype of HHT is not possible (Takaku et al., 1998). Even an endothelium specific SMAD4 deletion is embryonically lethal, as it shows angiogenic as well as cardiac defects (Lan et al., 2007; Qi et al., 2007).

The most recent HHT mutation identified lies within the gene for BMP9. Surprisingly, BMP9 knock-out mice develop normally and do not show any vascular defects (Chen et al., 2013). This lack of phenotype is most likely due to rescue by the closely related BMP10. Interestingly, injection of BMP10 neutralizing antibody into the BMP9 knock-out mice reduced the expansion of the retinal vasculature (Ricard et al., 2012; Chen et al., 2013). This shows that removal of both BMP9 and BMP10 ligands is necessary to induce vascular abnormalities.

In conclusion, mice heterozygous for endoglin or endothelium specific deletion of endoglin resembling HHT-1 (Arthur et al., 2000; Choi et al., 2012, 2014) and animals heterozygous for ALK1 mice resembling HHT-2 (Srinivasan et al., 2003), are suitable mouse models to unravel the etiology and mechanism behind HHT. Whether or not the double knock-out for BMP9/BMP10 will resemble a HHT subtype still needs to be established.

## Endoglin Expression on MNC and its Implications for Inflammatory and Regenerative Properties

As mentioned, endoglin and ALK1 are not only expressed on endothelial cells, but also on some subsets of the MNC fraction. The MNC fraction is an essential cell population during the inflammatory response and the repair process of damaged tissue. The MNC fraction consists of numerous different cell types with highly adaptive responses and cell plasticity. The most predominant cell types within the MNC fraction are T lymphocytes, monocytes, and macrophages. Furthermore, there are several smaller cell populations present, such as natural killer cells, dendritic cells and endothelial progenitor cells (EPCs; Isner et al., 2001).

In healthy subjects, endoglin is upregulated in activated monocytes, but this is impaired in HHT-1 patients (Sanz-Rodriguez et al., 2004). Interestingly, the increased expression of endoglin on activated monocytes was also impaired in HHT-2 patients (Sanz-Rodriguez et al., 2004). We propose that impaired signaling via endoglin and subsequent ALK1 signaling in MNC, and especially monocytes, might be causing immunological problems such as increased infection rate and leukopenia as reported (Guilhem et al., 2013; Peter et al., 2014).

### Two Phases of MNC Recruitment during Tissue Repair

**FIGURE 1:**
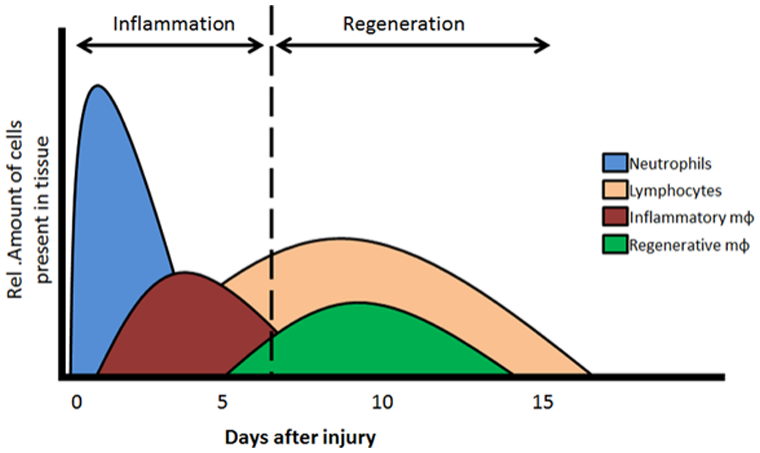
**Two phases of MNC recruitment**. In the acute phase (day 1–4) of tissue injury acute inflammation is initiated by the recruitment of neutrophils, monocytes and inflammatory type macrophages. Resolution of the inflammatory response in regeneration phase is elicited by the recruitment/dedifferentiation of regenerative monocytes/macrophages and lymphocytes. Mφ, macrophages. Adapted from Nahrendorf et al. (2007) and Loebbermann et al. (2012).

During tissue repair, two phases are essential, the inflammation and the regeneration phase. The MNC, and especially the monocytes and macrophages, play important and distinct roles in these two phases (**Figure 1**).

The first inflammatory phase is known as the acute phase, characterized by the infiltration of neutrophils, T lymphocytes and activated monocytes differentiating into inflammatory macrophages (**Figure 1**). T lymphocytes and inflammatory macrophages are necessary for the clearance of any infectious organisms and the removal of dead tissue and cell debris after an ischemic event and/or injury. The second regenerative phase is initiated when the initial influx of inflammatory cells is (partly) cleared from the site of injury. Secreted cytokines and growth factors stimulate a secondary phase of MNC and resident cells toward the regeneration area. Locally, TGFβ levels are elevated which will activate local endothelial cells and stimulate proliferation and repair/replace damaged vessels. Furthermore, the remaining MNCs, including a second type of monocytes that differentiate toward regenerative macrophages, are recruited which will shift the microenvironment toward tissue repair. The regenerative macrophages are able to induce cell proliferation, angiogenesis, and tissue remodeling. For an optimal resolution and recovery of damage tissues, both the inflammatory as well as regenerative macrophages are essential players. To note, macrophages have a high plasticity and are able to change between phenotypes, depending on their microenvironment (Kim and Hematti, 2010; Sindrilaru et al., 2011; Mantovani et al., 2013). Furthermore, macrophages have the capacity to interact with lymphocytes, as well as interact with and influence the viability and growth of mesenchymal stem cells and progenitor cells (Freytes et al., 2012). In addition, recent studies demonstrated that TGFβ stimulates the proliferation of regenerative macrophages, inducing a pro-fibrotic phenotype (Murray et al., 2011).

### MNC and Inflammation in HHT

The composition of the MNC fraction is different in HHT patients compared to healthy subjects. The amount of peripheral blood NK and T lymphocytes was found to be reduced in HHT-1, HHT-2, but also in the unidentified subtypes of HHT patients, while the B lymphocyte and monocyte populations were unaffected, including the phagocytic activity of the monocytes (Guilhem et al., 2013). Furthermore, TGFβ and endoglin have been shown to be essential factors during inflammation and tissue repair (Shull et al., 1992; Kulkarni et al., 1993; Larsson and Goumans, 2001; Ishida et al., 2004; Doetschman et al., 2012). As a consequence of these observations, HHT-1 and HHT-2 patients show an increased infection rate and leukopenia (Mathis et al., 2012; Guilhem et al., 2013), revealing an important role for disturbed inflammatory responses in HHT.

Since a role for ALK1 signaling in MNCs is less profound, we will focus on the function of endoglin in inflammatory responses. A role for endoglin in inflammatory disease became evident when Torsney et al. (2002) studied the expression of endoglin in human tissue samples and during wound healing. First, tissue sections taken from various affected organs (bowel, liver, and skin) in diseases such as inflammatory bowel disease, liver cirrhosis and granuloma showed that endothelial endoglin expression was highly upregulated, and correlated with inflammatory cell infiltrate, including lymphocytes and macrophages, in the immediate surrounding tissue (Torsney et al., 2002). Second, tissue sections from, e.g., skin lesions showed that there was a strong increase of endoglin 1–2 days after wounding, and a high level of endoglin persisted for up to 1 month. Interestingly, the peak of endothelial endoglin gene expression was reached at day 4, with the subsequent peak of protein expression at day 7, which coincides with the highest influx of inflammatory cells during the first week after tissue injury, also known as the acute inflammatory phase (Nahrendorf et al., 2007 and **Figure 1**).

In endoglin heterozygous mice, the restoration of a myocardial infarction was disturbed compared to wild type mice. This was characterized by a reduced cardiac function and by impaired vascularization of the damaged tissue. Interestingly, injection of human MNCs isolated from healthy volunteers into the circulation of the mice after myocardial infarction improved cardiac output and restored angiogenesis, while MNCs from HHT-1 patients did not have this effect (van Laake et al., 2006). Further analysis revealed that there were significant lower numbers of MNC of HHT-1 patients at the site of injury after myocardial infarction when compared to control MNC (van Laake et al., 2006; Post et al., 2010). In a similar study analyzing the kidneys, a reduced number of migrated MNC and macrophages was observed in endoglin heterozygous mice (Docherty et al., 2006). In conclusion, HHT patients have altered subsets of MNC and the differential expression of endoglin can explain the disturbance in their inflammatory response.

## Recruitment of MNC: Homing

**FIGURE 2:**
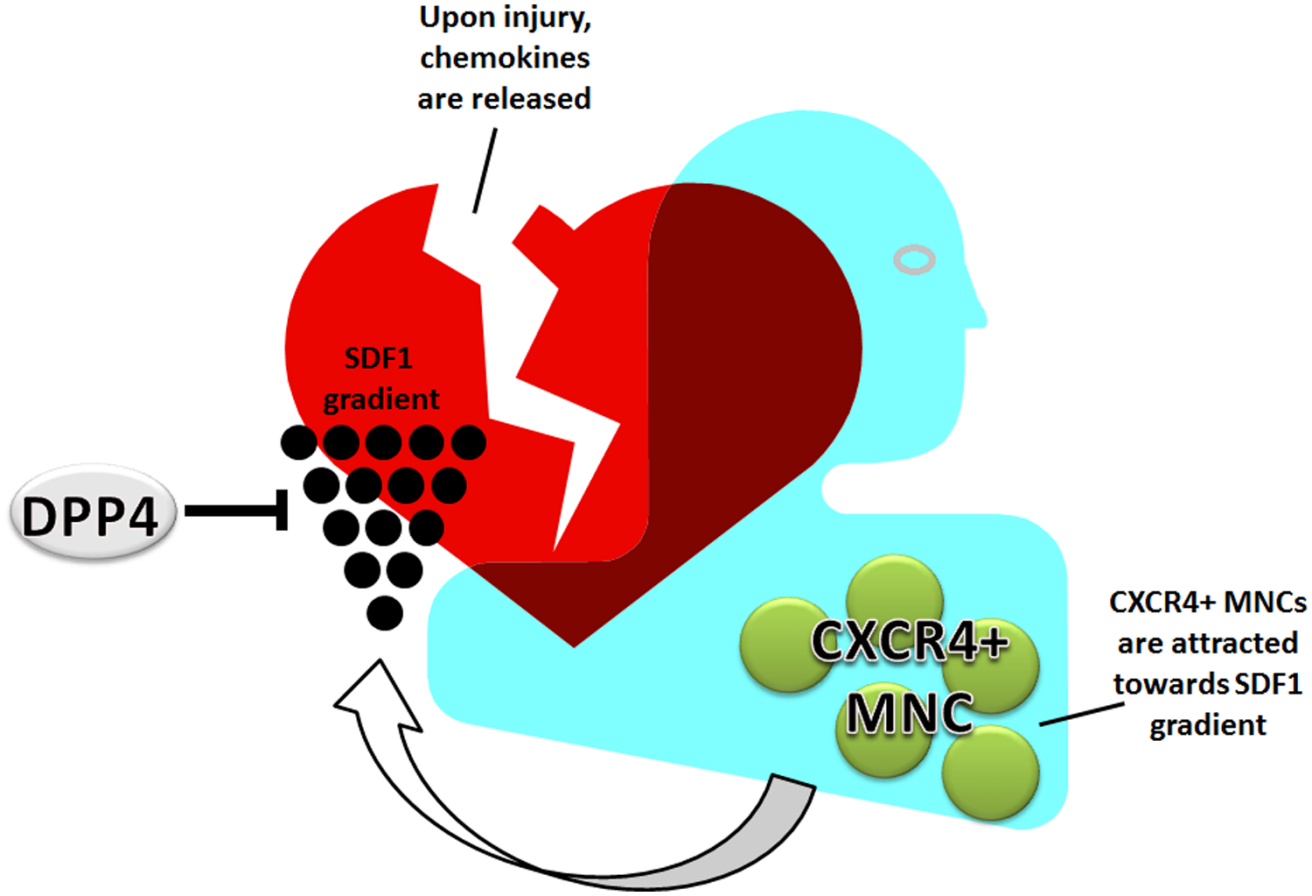
**Homing of MNC: the SDF1-CXCR4 axis**. MNC circulate through the vasculature or are retained by high SDF1 in bone marrow and spleen. Upon ischemia or tissue damage, SDF1 is released into the bloodstream creating an attracting gradient for MNC. The MNC home toward the damaged tissue via this gradient. This process is negatively regulated by DPP4.

Mononuclear cell can either be recruited from different sites, such as the spleen, bone marrow or they are already resident in tissue or in the blood. The process by which MNC, and also stem cells, are attracted to sites of ischemia or inflammation is tightly regulated. The main cellular homing mechanism is the SDF1-CXCR4 axis (**Figure 2**). MNC are retained in the bone marrow and spleen due to the high SDF1 levels (Ceradini et al., 2004). During ischemic disease, such as coronary artery, cerebrovascular, or peripheral artery disease, levels of SDF1 are increased. The occlusion of an artery results in hypoxia and an increase in hypoxia inducible factor 1 alpha (HIF1α) levels. HIF1α induces the expression of SDF1, which is then released into the bloodstream creating a gradient of SDF1, causing HSC and MNC to be recruited from the bone marrow and spleen, and migrate toward the highest SDF1 concentration present in the damaged tissue (Cencioni et al., 2012). A disturbed homing balance may result in increased fibrosis and adverse remodeling. For example, sustained activation of the SDF1-CXCR4 axis is observed in lung vessels of idiopathic pulmonary fibrosis patients (Smadja et al., 2014) and it was suggested that circulating fibrocytes contribute to intense remodeling of the pulmonary vasculature. Furthermore, infiltration of leukocytes can be mediated by integrins interacting and binding to endoglin, which could well be diminished in HHT-1 patients (Rossi et al., 2013).

Besides hypoxia, BMP9 is also a potent inducer of SDF1. The effects of BMP9 on SDF1 have been extensively studied in endothelial cells. Surprisingly, knockdown of either endoglin or ALK1 were shown to impair the upregulation of SDF1 by BMP9 (Young et al., 2012). Mutations in BMP9 are thus likely to decrease SDF1 levels, resulting in an impaired MNC homing capacity. In conclusion, impaired homing of MNC and impaired SDF1 regulation are a common feature of HHT.

In contrast, in other vascular diseases like atherosclerosis it has been described that enhanced homing of MNCs is part of the pathology. In atherosclerosis, low density lipoproteins (LDLs) increase endothelial SDF1 levels at the distal ends of the atherosclerotic plaques. In addition, LDL-induced SDF1 expression increase monocyte homing* in vitro* and monocyte adhesion via ICAM-1 interaction to the endothelium was enhanced and accelerated the progression of atherosclerotic plaque formation (Wei et al., 2012).

Therefore we conclude that homing via the SDF1-CXCR4 axis is delicate and skewing of either one of the proteins or regulators involved can cause defective inflammatory response and tissue repair.

## Regulation of MNC Recruitment by DPP4

The recruitment of MNC to sites of tissue damage is tightly regulated. In homeostasis, a negative regulator dipeptidyl peptidase 4 (DPP4) is able to prevent an uncontrollable infiltration of MNC. DPP4, also known as CD26, is a 110 kd transmembrane protein expressed by many different cell types, including endothelial cells, epithelial cells, melanocytes, monocytes, and lymphocytes (Yamada et al., 2009; Zhong et al., 2013). DPP4 is a peptidase that will enzymatically remove aminoterminal dipeptides after a proline or an alanine from specific proteins such as SDF1, neuropeptide Y or glucagon-like-protein 1. DPP4 also has non-enzymatic functions. It can influence T lymphocyte function by binding to adenosine deaminase, and acts as a stimulatory factor in T lymphocyte activation pathways (Yamada et al., 2009). Furthermore, DPP4 can interact and co-internalize with CXCR4 (Christopherson et al., 2002), again inhibiting the SDF1-CXCR4 axis.

The presence of DPP4 on the MNC cell membrane and target tissue, as well as the presence of the soluble form in plasma will influence the migration and recruitment of MNC. How soluble DPP4 is produced is not completely understood. One study suggests that soluble DPP4 is formed by shedding DPP4 from the cell membrane, while another study suggest that DPP4 is secreted by either liver epithelium or lymphocytes (Cordero et al., 2009; Wang et al., 2014b). The soluble form of DPP4 only lacks the intracellular and transmembrane parts, but keeps its enzymatic function (Lambeir et al., 2003).

Enhanced DPP4 activity will result in enhanced cleavage of SDF1, resulting in loss of its chemotaxic function. The cleavage of SDF1 therefore effectively puts a brake on the homing signal (Christopherson et al., 2002). SDF1 has three isoforms, SDF1α, β, and γ. While the functions of SDF1α and β are similar (Shioda et al., 1998), SDF1γ has a higher affinity for the binding protein for chemokines, heparan sulfate (Rueda et al., 2012). Heparan sulfate protects SDF1 against proteolysis induced by DPP4, keeping the homing signal intact. SDF1γ was found primarily expressed in the adult mouse heart and to a lesser extent in the brain (Torres and Ramirez, 2009). However, this restriction of organ expression in mice might not be similar in humans or during disease as SDF1γ was found upregulated in synovial dendritic cells and endothelial cells in patients with rheumatoid arthritis (Santiago et al., 2012). The role of SDF1γ in other diseases and the effects of decreased sensitivity to DPP4 are still unknown. However, increasing the expression of SDF1γ may be an option to stimulate the homing process in HHT-1 patients, making it a very interesting topic for future research.

## Alternative Homing Pathways and Mechanisms

Although the SDF1-CXCR4 axis is the main pathway, there are other mechanisms that influence the homing and the mobilization of MNC. For example, SDF1 is capable of binding to an alternative chemokine receptor, CXCR7. CXCR7 functions as a SDF1 receptor and increases MNC survival and adhesion (Döring et al., 2014). Furthermore, CXCR4 is able to bind to macrophage migration inhibitory factor (MIF). MIF is upregulated after MI, and specifically increases monocyte homing by competing with SDF1 for binding to CXCR4.

One other factor that influences homing is interferon-inducible protein 10 (IP10 or CXCL10). This peptide is secreted by a wide variety of cells, including MNC, fibroblasts and endothelial cells. IP10 functions as a lymphocyte chemotaxic cytokine after binding to its receptor CXCR3. Besides stimulating homing, IP10 can also induce migration and proliferation of endothelial cells and vascular smooth muscle cells (van den Borne et al., 2014). The decreased numbers of lymphocytes found in HHT patients (Guilhem et al., 2013) together with the impaired resolution of inflammation (Peter et al., 2014), suggest upregulation of IP10 might be another strategy to increase homing of MNC in HHT patients and restricting the inflammatory response.

The sympathetic nervous system is another pathway involved in the stimulation of homing and modulation of inflammatory responses. Wang et al. (2014a) showed that after stroke, β3-adrenergic receptor activity reduces the expression of SDF1 in the bone marrow, while the expression of CXCR4 was increased in bone marrow cells. The activated β3-adrenergic receptor also increased the levels of prostaglandin E2 in the bone marrow, which in turn mediates T lymphocyte activation via RANKL (Wang et al., 2014a). Moreover, cationic lipids such as C3a, anaphylatoxin and cathelicidin increase cell responsiveness to low SDF1 gradients, so-called ‘priming’ (Ratajczak et al., 2012).

In summary, the body has several mechanisms to respond to stress signals, resulting in a rapid and increased mobilization of MNC into the bloodstream followed by homing to the site of injury. The SDF1-CXCR4 axis is the most prominent and is malfunctioning in HHT-1 patients. Stimulating one of the other pathways may correct the homing deficiency present in HHT patients.

## DPP4 Inhibition in Type II Diabetes Mellitus and Cardiovascular Disease

Dipeptidyl peptidase 4 inhibitors like Sitagliptin, Vildagliptin, and Saxagliptin are currently in use to treat patients with type 2 diabetes mellitus (T2DM). Already at baseline, serum DPP4 levels are higher in T2DM patients compared to controls. DPP4 inhibition reduces the cleavage of glucagon-like-peptide 1, an incretin protein that is released upon food intake to decrease insulin levels (Mentlein et al., 1993; Deacon et al., 1998). Interestingly, T2DM patients show a decrease in progenitor cell mobilization (including EPC) from the bone marrow to the circulation – comparable to the impaired homing defect found in HHT. That DPP4 inhibition is a feasible treatment modality for the improvement of MNC homing is strengthened by the observation that in DPP4 deficient mice the mobilization capacity of the progenitor cell population was restored after myocardial infarction, and angiogenesis improved (Zaruba et al., 2009). Interestingly, high serum DPP4 levels were found to be associated with the occurrence of left ventricular dysfunction in T2DM patients (Ravassa et al., 2013). Furthermore, DPP4 inhibition can affect cardiomyocyte metabolism by restoring their ability to switch back to fatty acid metabolism during stress (Witteles et al., 2012).

There are many more actions of DPP4 where inhibition is capable of having protective effects in cardiac ischemia-reperfusion injury (Matheeussen et al., 2012). First, DPP4 is able to cleave the vasoconstrictor neuropeptide Y, stimulating angiogenesis via the eNOS pathway. Second, brain natriuretic peptide (BNP) is associated with congestive heart failure and is upregulated after ischemia (Mishra et al., 2014; Santaguida et al., 2014). BNP is cleaved by DPP4, providing protective effects through decreased natriuresis and vasodilation (Vanderheyden et al., 2009). Furthermore, DPP4 has a collagen binding domain – and consequently decreases collagen and fibronectin production, and thereby has the potential to decrease fibrosis (Thielitz et al., 2007). Thus, inhibiting DPP4 has a beneficial effect on tissue repair in more ways than only stimulating the SDF1-CXCR4 axis.

Dipeptidyl peptidase 4 inhibition using Sitagliptin has no effect on MNC subsets in healthy individuals (Price et al., 2013), while in disease the effects of DPP4 inhibition on MNC migration are profound. In both wild type mice and mice with induced continuously proliferating cardiomyocytes, combining DPP4 inhibition with granulocyte colony-stimulating factor (G-CSF) increased stem cell mobilization and stimulated myocardial repair (Zaruba et al., 2009, 2012; Theiss et al., 2011, 2013), not only via increased retention in the ventricular wall, but also via reduction of the adverse remodeling and enhanced angiogenesis. Patients who recover from a myocardial infarction express high DPP4 levels on their MNCs, which is associated with a decreased heart function (Post et al., 2012). DPP4 inhibition is therefore also an interesting treatment option for improving cardiac recovery. The first meta analyses using DPP4 inhibition in clinical trials show no adverse reactions and even a reduction of cardiovascular risks in T2DM patients treated with DPP4 inhibitors (such as Alogliptin, Vildagliptin, Sitagliptin, Saxagliptin, or Linagliptin; Monami et al., 2013; Avogaro et al., 2014). In contrast, DPP4 inhibition causes an increased prothrombogenic status of endothelial cells and correlates with upregulated tissue factor, the initiator of the coagulation cascade (Krijnen et al., 2012). This implicates that care should be taken, i.e., treatment may not always be beneficial to patients with increased coagulation status. Nonetheless, the overall data implicate that DPP4 inhibition has positive effects on tissue repair and subsequent cardiovascular function.

## DPP4 Inhibition in HHT

Interestingly, the expression of DPP4 is increased on MNCs from HHT-1 patients and might explain the disturbed homing of MNC and impaired tissue repair (van Laake et al., 2006; Post et al., 2010). In a follow up study the MNCs of HHT-1 patients were pretreated with a DPP4 inhibitor, which restored the amount of cells present at the site of infarct (Post et al., 2010). This study again suggests that not only the endothelial cells are affected in HHT-1, but also that the immune cells are involved. How MNCs are specifically affected by the disrupted TGFβ signaling in HHT patients is still not clear. However, several studies point toward a direct link between endoglin and the migratory capacity and function of MNC (Torsney et al., 2002; Post et al., 2010). *In vitro* studies also demonstrated a possible direct link between TGFβ and DPP4. For example, the inhibition of DPP4 increases the expression of TGFβ in MNC (Reinhold et al., 1997; Arndt et al., 2000), but induces a reduction of TGFβ expression in skin fibroblasts (Thielitz et al., 2007). In contrast, TGFβ is able to downregulate DPP4 expression in MNC and to reduce the numbers of DPP4+ cells. In HTT-1 patients this effect was less profound, but the DPP4 concentration on the cells is greatly reduced (Post et al., 2010, 2012).

How DPP4 might interfere with TGFβ signaling and vice versa is not clear. It was suggested that DPP4 has a co-receptor function with CD2 and CD3 on T lymphocytes, providing a possible direct way of interacting with TGFβ/endoglin signaling on the cell membrane (Morimoto and Schlossman, 1998; Gorrell et al., 2001).

## Clinical Perspectives and Conclusion

Hereditary hemorrhagic telangiectasia is not only a disease that affects the endothelial cells, the MNCs are also affected. The recruitment of MNC is impaired, due to increased levels of DPP4 and reduced levels of endothelial membrane endoglin. The inhibition of DPP4 in other vascular diseases shows beneficial results not only on the homing toward damaged tissues, but also on the recovery of functioning of the targeted tissue. For HHT, we know that DPP4 inhibition improves the homing of MNC. Its impact on actual tissue repair is unknown, and intriguing for future research. DPP4 inhibition will potentially restore homing and stimulate angiogenesis and tissue repair in HHT. However, this only holds true when the function of MNC are not impaired, e.g., improved homing of inflammatory macrophages will hamper the regenerative process. More knowledge on the homing and functioning of MNC can therefore contribute to develop and improve new therapeutic strategies for HHT.

## Conflict of Interest Statement

The authors declare that the research was conducted in the absence of any commercial or financial relationships that could be construed as a potential conflict of interest.
